# Structural basis for nuclear import of adeno-associated virus serotype 6 capsid protein

**DOI:** 10.1128/jvi.01345-24

**Published:** 2024-12-18

**Authors:** Mikayla Hoad, Sepehr Nematollahzadeh, Gayle F. Petersen, Justin A. Roby, Gualtiero Alvisi, Jade K. Forwood

**Affiliations:** 1 School of Dentistry and Medical Sciences, Charles Sturt University 376290 , Wagga Wagga, New South Wales, Australia; 2 Gulbali Institute, Charles Sturt University 72531 , Wagga Wagga, New South Wales, Australia; 3 Department of Molecular Medicine, University of Padova 208970 , Padova, Italy; International Centre for Genetic Engineering and Biotechnology, Trieste, Italy

**Keywords:** nuclear import, adeno-associated virus, importin, karyopherin

## Abstract

Adeno-associated viruses (AAVs) are the most extensively researched viral vectors for gene therapy globally. The AAV viral protein 1 (VP1) N-terminus controls the capsid’s ability to translocate into the cell nucleus; however, the exact mechanism of this process is largely unknown. In this study, we sought to elucidate the precise interactions between AAV serotype 6 (AAV6), a promising vector for immune disorders, and host transport receptors responsible for vector nuclear localization. Focusing on the positively charged basic areas within the N-terminus of AAV6 VP1, we identified a 53-amino acid region that interacts with nuclear import receptors. We measured the binding affinities between this region and various nuclear import receptors, discovering a notably strong interaction with IMPα5 and IMPα7 in the low nanomolar range. We also elucidated the X-ray crystal structure of this region in complex with an importin alpha (IMPα) isoform, uncovering its binding as a bipartite nuclear localization signal (NLS). Furthermore, we show that using this bipartite NLS, AAV6 VP1 capsid protein can localize to the nucleus of mammalian cells in a manner dependent on the IMPα/IMPβ nuclear import pathway. This study provides detailed insights into the interaction between the AAV6 VP1 capsid protein and nuclear import receptors, deepening our knowledge of AAV nuclear import mechanisms and establishing a basis for the improvement of AAV6-based gene therapy vectors.

**IMPORTANCE:**

AAVs, recognized as the most extensively researched viral vectors for gene therapy globally, offer significant advantages over alternatives due to their small size, non-pathogenic nature, and innate ability for tissue-specific targeting. AAVs are required to localize to the nucleus to perform their role as a gene therapy vector; however, the precise mechanisms that facilitate this process remain unknown. Despite sharing overt genomic similarities with AAV1 and AAV2, AAV6 is a unique serotype. It is currently recognized for its ability to effectively transduce hematopoietic cell lineages and, consequently, is considered promising for the treatment of immune disorders. Identifying the exact mechanisms that permit AAV6 to access the nucleus can open up new avenues for gene therapy vector engineering, which can ultimately lead to increased therapeutic benefits.

## INTRODUCTION

Adeno-associated viruses (AAVs) were originally discovered in 1965 as a contamination of adenovirus preparations (1) and over the last ~50 years have become one of the world’s most extensively studied viral gene therapy vectors (2), with three Food and Drug Administration-approved, AAV vector-derived therapeutics currently available for treating genetic disorders (Glybera, Luxturna, and Zolgensma) (3). Members of the family *Parvoviridae* and the genus *Dependoparvovirus* (4, 5), AAVs are small (~4.7 kb), single-stranded DNA viruses containing three genes (*rep*, *cap*, and *aap*) flanked by inverted terminal repeats (6–8). There are at least 12 recognized AAV serotypes isolated from human and primate tissues (9), with over 150 different serotype variants. More than 80% of the human population is seropositive for at least one form of wild-type AAV (10–13), but infection is largely asymptomatic due to their limited capacity to invoke the immune response, incapability to replicate without co-infection of the host with another virus (14, 15), and an inability to cause physical disease manifestations (16).

AAVs are considered a revolutionary target for gene therapy with the potential to address a wide array of genetic disorders and diseases. Their natural tropism for human cells (17), ability to infect both non-dividing and dividing cells (18–20), and minimal immunogenicity (21, 22) are all huge positives for AAV vector use. This, coupled with a small genome that can be easily modified to carry therapeutic transgenes (23) and persistent expression of the transgene in cells (24), further expands the potential for use of AAVs as gene therapy vectors. In addition to these benefits, there are a number of drawbacks associated with the use of AAV vectors in gene therapy. These include a small therapeutic transgene size allowance, pre-existing antibodies which result in a loss of efficiency in some patients, administration of AAV vectors at high doses leading to toxicity, and the overall cost and complexity of viral vector manufacturing. Considering the ease of AAV capsid modification, we have found that identifying a target for modification that could assist in creating a better and more efficient therapeutic vector that could be used at a lower dose without restructuring or redesigning the entirety of the capsid virion would be ideal.

Though the conceptual understanding of the ability of AAVs to localize into the cell nucleus is well known, the exact mechanisms and interactions with host proteins that mediate this translocation remain to be fully explored for human serotypes. Until recently, studies have only shown that AAV utilizes the nuclear pore complex (25–27), identified potential regions of the capsid which facilitate nuclear translocation (28), and shown that AAV can interact in some manner with importin beta (IMPβ) receptor proteins (29). Considering the importance of AAV translocation into the nucleus for gene therapy, we have found that understanding the mechanisms mediating this process would not only expand our general knowledge but also allow for vector improvement. We have recently elucidated the direct binding interactions between different AAV serotypes/variants, porcine and bat AAV strains, and host nuclear import receptor proteins (30–32). Our research also shows that AAV interacts with importin alpha (IMPα) proteins but not directly with IMPβ proteins as proposed by others (29), suggesting that AAVs utilize the classical nuclear import pathway.

The classical nuclear import pathway involves the binding of cargo proteins to the host import receptor protein IMPα via a nuclear localization signal (NLS) (33). IMPα binds with an additional import receptor protein, IMPβ1, forming a trimeric complex which translocates across the nuclear envelope via the nuclear pore complex (34). Once inside the nucleus, the complex is dissociated by RanGTP. IMPα and IMPβ1 are recycled back to the cytoplasm for future use, and the cargo is left to perform its role within the nucleus (35). A critical mechanism of the classical pathway is the interaction between the cargo’s NLS, serving as a recognition motif, and a host IMPα (36, 37). An NLS is a short sequence of positively charged amino acids, comprising either one region, which interacts with one binding site of IMPα (monopartite NLS) (37–40), or two regions, separated by a short “linker,” which interact concurrently with two binding sites of IMPα (bipartite NLS) (33, 41–43).

The AAV *cap* gene encodes three viral capsid proteins of different lengths (viral protein 1 [VP1], VP2, and VP3), all of which share a C-terminal domain but have different lengths of the N-terminal region. VP1 contains the entire N-terminal domain (44–46); VP2 is slightly shorter by ~140 residues (44–46); and VP3 does not retain any of the N-terminal domain (44–46). When VP1, VP2, and VP3 interact to form the outer capsid shell of the AAV virion, it is suspected that the flexible N-terminal regions of VP1 and VP2 sit within the capsid shell (47, 48) and become exposed before nuclear localization (49–54). Previous data suggest that there are specific basic regions (BRs) within the N-terminal domain that likely act as NLSs (28), with mutations in these regions reducing the nuclear import efficacy of full-length VP proteins (29, 55) and limiting recombinant AAV capsid interactions with IMPβ1 proteins (29). More recent data have validated that these regions of VP1/VP2 act as NLSs and interact with IMPα proteins (30–32). The porcine AAV VP1-BR, which shares a near-identical BR1 sequence with all human and non-human primate AAVs, was shown to bind all IMPα isoforms but not IMPβ1, with BR1 interacting with the IMPα minor binding site and an alternate sequence downstream of BR1 (similar to that seen in AAV5 VP1) interacting with the IMPα major binding site (31). The bat AAV VP1-BR, with near-identical BR1 and BR3 sequences to majority of human serotypes, similarly bound all IMPα isoforms but not IMPβ1, with BR3 interacting with the major binding site and an alternate BR2 region interacting with the minor binding site of IMPα (30). The lack of interactions with IMPβ1 strongly suggests a need for IMPα to act as an adaptor protein to utilize the classical nuclear import pathway.

AAV serotype 6 (AAV6) is thought to be a hybrid recombinant between AAV serotype 1 and AAV serotype 2; however, it has kept its serotype numbering due to the difference in serotype transduction profile. This is suspected to be a result of differences in both cellular uptake and intracellular trafficking mechanisms of AAV serotype vectors, in particular, the structural differences at a capsid level (9). Currently, AAV6 has been shown to transduce lymphocytes (56–58) and has been used in the production of chimeric antigen receptor T cells for immunotherapeutics (59–65). Although AAV6 VP1 only shares ~57% and ~60% amino acid sequence identity with the above-mentioned porcine and bat AAV VP1 proteins, respectively, BR1 is conserved and BR3 only deviates by a single residue between AAV6 (^165^QPA**K**KRL^170^) and bat AAV (^160^QPA**R**KRL^165^). Considering the AAV6 VP1 N-terminal BR is near identical to those of AAV1 and AAV2, understanding the mechanism behind nuclear transport and the involved residues can shed light not only on AAV6 but also on AAV1 and AAV2.

This study has focused on understanding the structural mechanisms between AAV6 VP1 and host nuclear import proteins to further understand the exact interactions driving AAV6 localization to the nucleus for transgene transduction and subsequent therapeutic protein expression. The AAV6 VP1-BR was shown to be unable to bind IMPβ1, binding strongly with IMPα isoforms and indicating that AAV6 VP1 uses the classical nuclear import pathway. Biochemical assays showed that the AAV6 VP1-BR bound to IMPα isoforms with varying specificities, exhibiting the strongest binding affinity for IMPα7. Crystallography studies demonstrated that the AAV6 VP1-BR possesses a classical bipartite NLS, with binding mediated through BR1 and BR3. For the very first time, cellular inhibition assays demonstrated that full-length AAV6 VP1 must utilize the classical IMPα/IMPβ-dependent nuclear import pathway. Of importance, it was also found that BR1 and BR3 are essential, as without these two BRs, nuclear localization of full-length AAV6 VP1 cannot occur efficiently. The residues of AAV6 VP1 driving IMPα binding interaction were identified and shown to be essential, enhancing the future capacity for vector engineering with this serotype.

## MATERIALS AND METHODS

### Gene construct design

The amino acid sequence of AAV6 VP1 protein was sourced using the GenBank database (accession AAB95450.1), and residues ^115^GRAVFQAKKRVLEPFGLVEEGAKTAPGKKRPVEQSPQEPDSSSGIGKTGQQPAKKRLNFG^174^ (referred hereafter as AAV6 VP1-BR) were codon optimized for expression in *Escherichia coli*. The gene was synthesized with an additional upstream N-terminal tobacco etch virus (TEV) protease site (for GST-tag cleavage) and cloned into the pGEX4T-1 vector at the BamHI sites (GenScript, USA). A pET30a expression vector was used to express mouse IMPα2, which lacks residues 1–70 that form the IMPβ1-binding domain (IBB), to improve overall expression and purification without being detrimental to the functionality of IMPα as a binding partner (IMPα2ΔIBB; His tag, no TEV site), as previously described (66).

As previously described, IMPs used for electromobility shift assays (EMSAs) and fluorescence polarization (FP) assays include human α1 (hIMPα1ΔIBB; His tag, TEV site), α3 (hIMPα3ΔIBB; His tag, TEV site), α5 (hIMP5ΔIBB; His tag, TEV site), α7 (hIMPα7ΔIBB; His tag, TEV site), and mouse α2 (IMPα2ΔIBB; His tag, no TEV site) in pET30a (66–68) and human β1 (hIMPβ1; His tag, TEV site) in pMCSG21 (69, 70).

Plasmids mediating the mammalian cell expression of C-terminal green fluorescent protein (GFP) fusions with full-length AAV6 VP1 protein, along with the respective mBR1+3 substitution derivative, were synthesized by GenScript. Plasmids pEPI-GFP-human cytomegalovirus (HCMV)-UL44 (71) and pEGFP-N1-FrAdV1 (72), encoding GFP fusion proteins which localize to the nucleus via IMPα/IMPβ-dependent and independent pathways, were described previously. Plasmid mcherry-Bimax2 (73), encoding for a competitive inhibitor of the IMPα/IMPβ nuclear import pathway (73, 74), was a generous gift from Yoshihiro Yoneda and Mashiro Oka (Osaka, Japan).

### Fluorescein isothiocyanate-labeled peptide design

A synthetic peptide corresponding to the AAV6 VP1-BR sequence and including an N-terminal Ahx-linker and fluorescein isothiocyanate (FITC) fluorescent tag was obtained through GenScript. The peptide spanned amino acid residues ^120^QAKKRVLEPFGLVEEGAKTAPGKKRPVEQSPQEPDSSSGIGKTGQQPAKKRLN^172^.

### Recombinant protein expression and purification

Expression and subsequent co-purification of AAV6 VP1-BR and IMPα2 were performed in a manner analogous to the previously described method using a porcine AAV NLS (31, 32).

For use in FP assays and EMSAs, overexpression of IMPα1, IMPα2, IMPα3, IMPα5, IMPα7, and IMPβ1 was performed in *E. coli*, and each recombinant protein was later purified using methods previously described (31, 32, 75).

### Crystallization of AAV6 VP1-BR and IMPα2 complex

The AAV6 VP1-BR and IMPα2 protein complex was crystallized using the hanging drop vapor diffusion method described previously (76), with a final crystallization condition of 0.7-M sodium citrate, 0.01-M dithiothreitol, 0.1-M HEPES, pH 7.5, mixed in a 1:1 ratio with a protein complex solution and incubated for 3 days at 22°C. Rod-shaped crystals (140 × 15 × 15 μm) were collected, cryoprotected in a reservoir solution containing 20% glycerol for 10 seconds, and flash frozen at −196°C in liquid nitrogen.

### Data collection and structure determination

X-ray diffraction data of the protein complex crystal was obtained on the MX2 beamline at the Australian Synchrotron (77). Data indexing and integration were performed using the program DIALS (78), and subsequent merging and scaling of data were completed using Aimless (79, 80). The structure 4OIH (68) from the Protein Data Bank was used as a search model for molecular replacement achieved using the program Phaser (81). Model rebuilding and refinement were achieved via programs Phenix (82) and Coot (83, 84). The final structure was solved to a resolution of 2.3 Å and refined to *R*_work_/*R*_free_ values of 0.22/0.24. The final model consisted of 426 residues of IMPα2, 15 residues of AAV6 VP1-BR, and 34 water molecules. The final stereochemistry and additional refinement statistics are presented in Table 1. All interactions and associated hydrogen bonds and salt bridges were determined using the database PDBsum (85), with calculations for hydrogen bonds performed by HBPLUS software (86), salt bridges using the definition by Kumar and Nussinov (87), and interface surfaces computed using the program NACCESS (88). All hydrogen bonds and salt bridges determined by the software are shown in Table 2.

**TABLE 1:** Data collection and refinement statistics for the structure of importin-α in complex with the AAV6 VP1 basic region (PDB code 9CFT)

Parameter	Value
Data collection	
Wavelength	0.9537
Data-collection temperature (K)	100
Detector type	DECTRIS EIGER X 16M
Detector	Pixel
Resolution range (Å) (°)	19.79–2.30 (2.382–2.3)
Space group	P 21 21 21
Cell lengths	79.01, 89.32, 101.64
Cell angles	90.00, 90.00, 90.00
Total observations	183,094 (16,836)
Unique observations	32,611 (3,161)
Multiplicity	5.6 (5.3)
Completeness (%)	99.8 (100.0)
Mean *I*/*σ* (*I*)	10.5 (2.0)
Wilson B-factor (Å^2^)	46
*R*_pim_	0.051 (0.739)
Refinement	
*R*_work_	0.22 (0.31)
*R*_free_	0.24 (0.31)
No. of non-hydrogen atoms	3,410
Macromolecules	3,378
Solvent	32
Total protein residues	441
Bond length r.m.s.d. (Å)	0.002
Bond angle r.m.s.d. (°)	0.50
Ramachandran favored (%)	98.17
Ramachandran allowed (%)	1.83
Ramachandran outliers (%)	0.00
Average B-factor	58.68
Macromolecules (overall)	58.81
Macromolecules (IMPα)	57.83
Macromolecules (AAV6 VP1-BR)	59.36
Solvent	45.37

**TABLE 2:** AAV6 VP1-BR hydrogen bond and salt bridge interactions with IMPα2

IMPα2	AAV6 VP1-BR
Hydrogen bonds	
TRP 142 [NE1]	ASN 172 [O]
ASN 146 [OD1]	ASN 172 [N]
ASN 146 [ND2]	ASN 172 [O]
GLY 150 [O]	LYS 169 [NZ]
THR 155 [OG1]	LYS 169 [NZ]
TRP 184 [NE1]	ARG 170 [O]
ASN 188 [OD1]	ARG 170 [N]
ASN 188 [ND2]	ARG 170 [O]
ASP 192 [OD1]	LYS 169 [N]
ASN 228 [OD1]	ARG 170 [NH1]
ASN 228 [OD1]	ARG 170 [NH2]
TRP 231 [NE1]	ALA 167 [O]
ASN 235 [ND2]	LYS 168 [O]
ARG 238 [NH1]	PRO 166 [O]
ARG 238 [NH2]	PRO 166 [O]
VAL 321 [O]	LYS 123 [NZ]
THR 328 [OG1]	LYS 123 [NZ]
SER 360 [OG]	ARG 124 [NH1]
ASN 361 [O]	LYS 123 [NZ]
ASN 361 [OD1]	ARG 124 [N]
GLU 396 [OE1]	ARG 124 [NH1]
Salt bridges	
ASP 192 [OD1]	LYS 169 [NZ]
GLU 396 [OE1]	ARG 124 [NH1]

### Electromobility shift assay

EMSAs were undertaken using a previously described protocol (30, 31). FITC-tagged AAV6 VP1-BR was used at 10 µM and mixed with importin isoforms (IMPα1, IMPα2, IMPα3, IMPα5, IMPα7, and IMPβ1) at 20 µM.

### Fluorescence polarization assay

FP assays were performed using a previously described protocol (30, 31). FITC-tagged AAV6 VP1-BR was used at 2 nM, and the importin isoforms (IMPα1, IMPα2, IMPα3, IMPα5, IMPα7, and IMPβ1) started serial dilutions at an upper concentration of 4.5 µM. Statistical analysis of the data was performed via one-way analysis of variance (ANOVA) using Tukey’s post-test for multiple comparisons of *K*_D_ binding values with GraphPad Prism version 9 software (GraphPad, San Diego, CA, USA).

### Cell culture and transfections

HEK 293A cells were cultured in Dulbecco’s modified Eagle’s medium (DMEM) with 10% (vol/vol) fetal bovine serum, 50 U/mL penicillin, 50 U/mL streptomycin, and 2 mM L-glutamine. The cells were kept in a humidified incubator at 37°C with 5% CO_2_ and were passaged upon reaching confluence. For transfection, HEK 293A cells were seeded onto glass coverslips in a 24-well plate (5 × 10^4^ cells/well). The following day, the cells were transfected with varying amounts of expression constructs (5–250 ng) using Lipofectamine 2000 (Thermo Fisher Scientific, Monza, Italy), according to the manufacturer’s instructions. The cells were then incubated at 37°C with 5% CO_2_ in complete medium (89) until they were ready for processing for confocal laser scanning microscopy (CLSM).

### Confocal laser scanning microscopy and image analysis

Twenty-four hours post-transfection, the cells were incubated for 30 minutes with DRAQ5 (#62251, Thermo Fisher Scientific; 1:5,000 in DMEM without phenol red). The cells were then washed twice with PHEM 1× buffer (60 mM PIPES, 25 mM HEPES, and 10 mM EGTA, and 4 mM MgSO_4_) and fixed with 4% (vol/vol) paraformaldehyde in PHEM for 10 minutes at room temperature. Following three washes with 1× phosphate-buffered saline, coverslips were mounted on glass slides using Fluoromount G (Southern Biotech, Birmingham, AL, USA). The subcellular localization of fusion proteins was analyzed using a Nikon A1 confocal laser scanning microscope (Nikon, Tokyo, Japan) equipped with a 60x oil immersion objective, as previously described (90, 91). Levels of nuclear accumulation of proteins of interest were determined using the FiJi software (92) from single-cell measurements of nuclear (Fn), cytoplasmic (Fc), and autofluorescence/background (Fb) fluorescence, following the subtraction using the formula Fn/c = (Fn − Fb) / (Fc − Fb), as previously described (93). Statistical analysis of the data was performed using the Welch and Brown-Forsythe ANOVA test with GraphPad Prism 9 software.

## RESULTS

### AAV6 VP1-BR interacts with all subfamilies of importin alpha proteins but not importin beta

**Fig 1:**
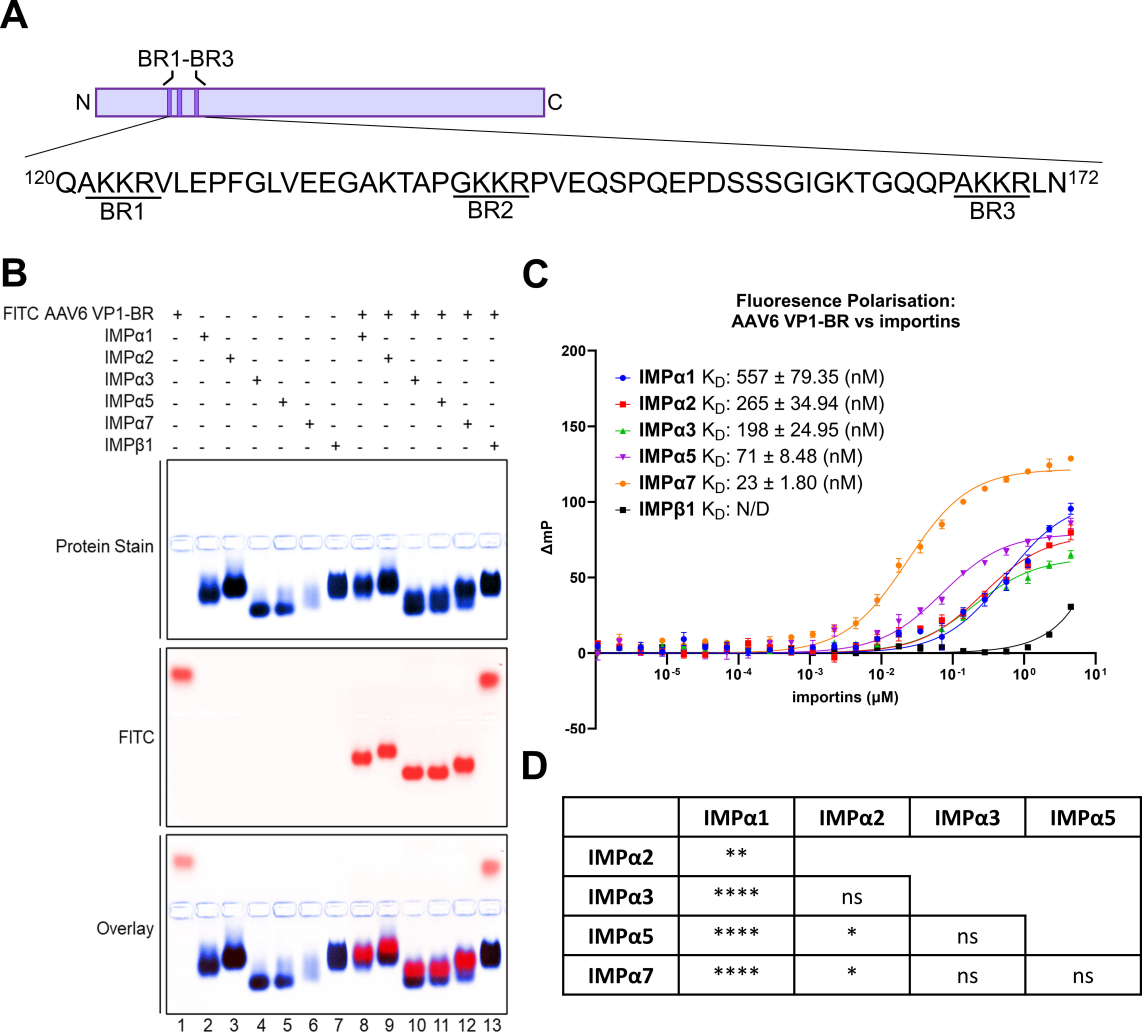
AAV6 VP1-BR binds to importin alpha isoforms but not importin beta 1. (**A**) AAV6 VP1-BR FITC-tagged peptide sequence used for EMSA and FP. The peptide spans residues 120–172 and contains an N-terminal FITC tag and Ahx linker. (**B**) EMSA showing AAV6 VP1-BR with ΔIBB-IMPα isoforms spanning members from each of the three subfamilies (SF1, IMPα1/IMPα2; SF2, IMPα3; SF3, and IMPα5/7) and IMPβ1. The FITC peptide is shown false colored in red (middle panel). Proteins were stained using Coomassie blue stain (top panel, blue). The overlay is represented at the bottom panel, where FITC peptide (red) overlays with protein (blue) to indicate co-migration (binding) of AAV6 VP1-BR with all IMPα proteins but not IMPβ1. EMSA results are representative of three independent experiments. (**C**) FP assay measuring the direct binding between AAV6 VP1-BR FITC peptide and respective importin isoforms. Binding was observed with IMPα1 (*K*_D_ = 557 nM), IMPα2 (*K*_D_ = 265 nM), IMPα3 (*K*_D_ = 198 nM), IMPα5 (*K*_D_ = 71 nM), and IMPα7 (*K*_D_ = 23 nM). Binding with IMPβ1 was so low that an accurate *K*_D_ could not be determined (N/D). Error bars were calculated using the standard error of the mean of three independent experiments. The error for the *K*_D_ values was the standard error of the mean. (**D**) Statistical results of a one-way ANOVA test using Tukey’s post-test for multiple comparisons of the FP *K*_D_ results. All *P* values of comparison between IMPα isoforms are displayed as follows: ns as *P* > 0.05; *P ≤ 0.05, **P ≤ 0.01, ****P ≤ 0.0001. ns, non-significant.

To investigate the capability of the N-terminal BR of AAV6 VP1 (Fig. 1A) to interact with nuclear import receptor proteins (and specifically whether it engages with IMPβ1 either directly or via IMPα adapters), we employed an EMSA. This approach was used to qualitatively identify variations in binding affinity of AAV6 VP1-BR for different importin isoforms (IMPα1, IMPα2, IMPα3, IMPα5, IMPα7, and IMPβ1). We found that the AAV6 VP1-BR bound to all IMPα isoforms (Fig. 1B), spanning across the three subfamilies: SF1 (IMPα1 and IMPα2), SF2 (IMPα3), and SF3 (IMPα5 and IMPα7). In contrast, AAV6 VP1-BR did not show binding to IMPβ1 (Fig. 1B), indicating a distinct and direct binding for IMPα.

To deepen our understanding of the interaction between AAV6 VP1-BR and IMP isoforms beyond qualitative measures, we quantified the binding affinities using FP (67). AAV6 VP1-BR exhibited moderate binding affinities for IMPα1 (557 nM), IMPα2 (265 nM), and IMPα3 (198 nM), and higher affinity toward IMPα5 (71 nM), with the highest affinity observed for IMPα7 (23 nM) (Fig. 1C). As such, AAV6 VP1-BR bound with the greatest affinity to the IMPα SF3 subfamily members, IMPα5 and IMPα7. Consistent with the EMSA findings, AAV6 VP1-BR’s affinity for IMPβ1 was so low that a dissociation constant could not be reliably measured (Fig. 1C), suggesting that AAV6 VP1-BR is unlikely to interact directly with IMPβ1 in a cellular environment.

### Structural analysis indicates that AAV6 VP1-BR interacts with IMPα through a bipartite nuclear localization signal

**Fig 2:**
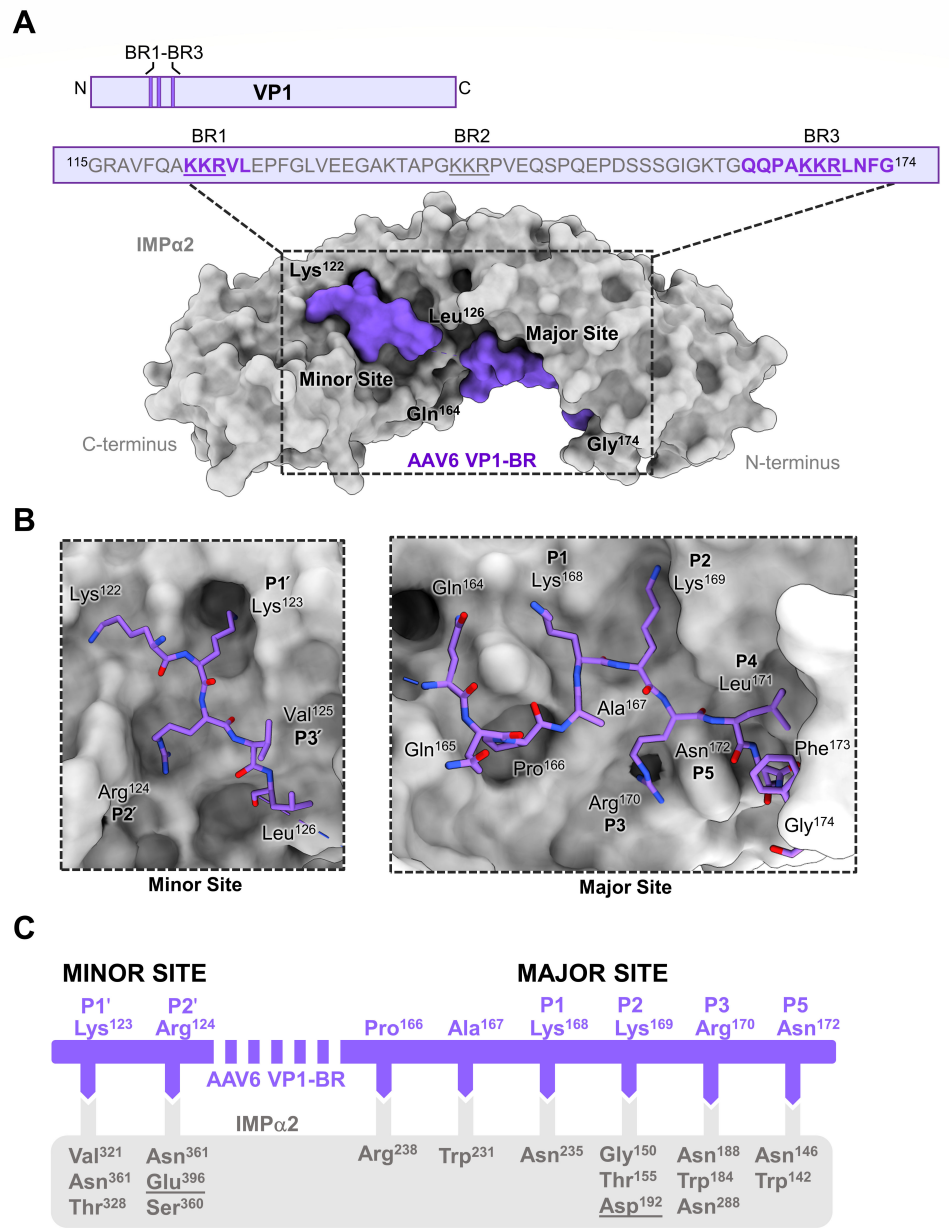
Crystal structure reveals AAV6 VP1-BR as a bipartite NLS in complex with IMPα2. (**A**) Schematic overview of the AAV6 VP1 protein and structure of the AAV2 VP1-BR (purple surface) and IMPα2 (gray surface) complex resolved to 2.3-Å resolution. The sequence of AAV6 VP1-BR bound to IMPα2 is detailed in the box (BRs determined by sequence alignment are underlined, with residues seen within the structure in bold purple, and residues not able to be resolved in the structure are denoted in gray). This structure has been deposited to the PDB and givPen the code 9CFT. (**B**) Major and minor binding sites of IMPα2 (gray surface) with labeled AAV6 VP1-BR residues (purple sticks) occupying sites and *P* positions indicated. (**C**) Simplified representation of IMPα2 and AAV6 VP1-BR binding interactions. The AAV6 VP1-BR (purple line) residues forming bonds with IMPα2 residues (gray box) are indicated through complementary arrows. Salt bridges are indicated via underlined IMPα2 residues, and non-underlined residues indicate hydrogen bonds. binding interactions were determined by the PDBsum server. P4 position Leu^171^ is not depicted as it does not form any hydrogen bonds or salt bridge interactions with IMPα2.

To elucidate the specific mechanism of interaction between AAV6 VP1 and IMPα, X-ray crystallography was utilized. Complexes of AAV6 VP1-BR with IMPα3, IMPα5, and IMPα7 were produced; however, crystals and thus structural data were unable to be obtained. As such, the structure of AAV6 VP1-BR was solved in complex with IMPα2 at 2.3-Å resolution. Although only a moderate binder, IMPα2 was utilized due to the ease in which crystals can be grown for high-resolution X-ray diffraction data. The IMPα2 structure exhibited the anticipated α-helical architecture across the 10 armadillo (ARM) motifs, consistent with prior descriptions (89). AAV6 VP1-BR interacted with IMPα2 at sites involving both the minor (ARMs 6–8, P1′–P3′) and major (ARMs 2–4, P1–P5) binding sites (Fig. 2). This interaction pattern is consistent with that of a bipartite NLS (68) (Table 3). In classical NLSs, residues form salt bridges and hydrogen bonds with strategically positioned, negatively charged residues that line the binding pockets of IMPα, and in our structure, a similar binding mechanism is employed (38, 94, 95). The observed interaction encompasses a buried surface area of 1,230 Å^2^, mediated by 6 hydrogen bonds and 1 salt bridge in the minor binding site, and 15 hydrogen bonds and 1 salt bridge in the major binding site of IMPα2 (see Table 2 for a full list of interactions).

**TABLE 3:** Amino acid residues (single letter representation) occupying the P site positions of viral protein classical NLSs of IMPα minor and major sites compared to AAV6 VP1-BR

PDB	Viral protein	P1′	P2′	P3′	Linker	P1	P2	P3	P4	P5	Reference
1EJL	SV40 T-antigen	K	K	R	–	K	K	K	R	K	(38)
7RG6	HKU5 ORF4b	K	R	K	–	R	K	R	R	R	(67)
8TUR	MCPyV LTA	K	R	K	–	P	K	K	N	R	(91)
8TUU	MWPyV LTA	K	R	P	–	P	K	R	P	R	(91)
8TUQ	STLPyV LTA	K	R	K	–	P	K	K	N	K	(91)
8TUS	WUPyV LTA	K	R	T	–	P	K	K	K	K	(91)
8Q8K	KIPyV LTA	K	R	S	–	P	K	K	K	-	(91)
6BVV	Nipah W protein	R	R	V	–	T	K	K	A	R	(96)
6BW9	Hendra W protein	R	R	V	–	T	K	K	A	R	(96)
9CFT	AAV6 VP1-BR	K	R	V	–	K	K	R	L	N	–

The major binding site of IMPα has a binding NLS consensus sequence of XK[K/R]X[K/R]X, where X can be any residue. Our structure follows this consensus, with the AAV6 VP1-BR BR3 residues 168-KKRLN-172 occupying the P1–P5 positions of IMPα (Fig. 2B and C). AAV6 VP1-BR featured a hydrogen bond from AAV6 VP1-BR Lys^168^ to IMPα2 Asn^235^ at the P1 binding site. The key P2 site interaction involved AAV6 VP1-BR Lys^169^ forming hydrogen bonds with IMPα2 Gly^150^, Thr^155^, and Asp^192^, alongside a salt bridge with Asp^192^. This P2 position Lys^169^ and its interactions with IMPα are canonical with all other major binding site classical NLSs. At the P3 site, AAV6 VP1-BR Arg^170^ forms hydrogen bonds with IMPα2 Asn^188^, Trp^184^, and Asn^288^. The designated P5 site displayed hydrogen bonds linking IMPα2 Asn^146^ and Trp^142^ to AAV6 VP1-BR Asn^172^. The details of these hydrogen bonds and salt bridge interactions are summarized in Table 2.

The minor binding site of IMPα2 interacts with the typical ‘KR’ motif of AAV6 VP1-BR BR1 residues 123-KR-124 (Fig. 2B and C). This is a common observation of bipartite NLSs, where the consensus binding sequence of the NLS is [K/R][K/R]XX, where X can be any residue (33, 38, 43, 97). Residue Lys^123^ of AAV6 VP1-BR interacted with IMPα2 residues Val^321^, Asn^361^, and Thr^328^ via hydrogen bonds to form the P1′ binding site. AAV6 VP1-BR residue Arg^124^ formed hydrogen bonds with Asn^361^, Glu^396^, and Ser^360^ of IMPα2, in addition to a salt bridge with Glu^396^ of IMPα2 as the P2′ binding site. Binding interactions are further detailed in Table 2. The linker region between BR1 and BR3 (residues 127–163) cannot be resolved within the structure, most likely due to a lack of strong interactions with IMPα and the overall flexibility of this region.

### AAV6 VP1 utilizes the classical import pathway in mammalian cells, requiring both BR1 and BR3 as a nuclear localization signal

**Fig 3:**
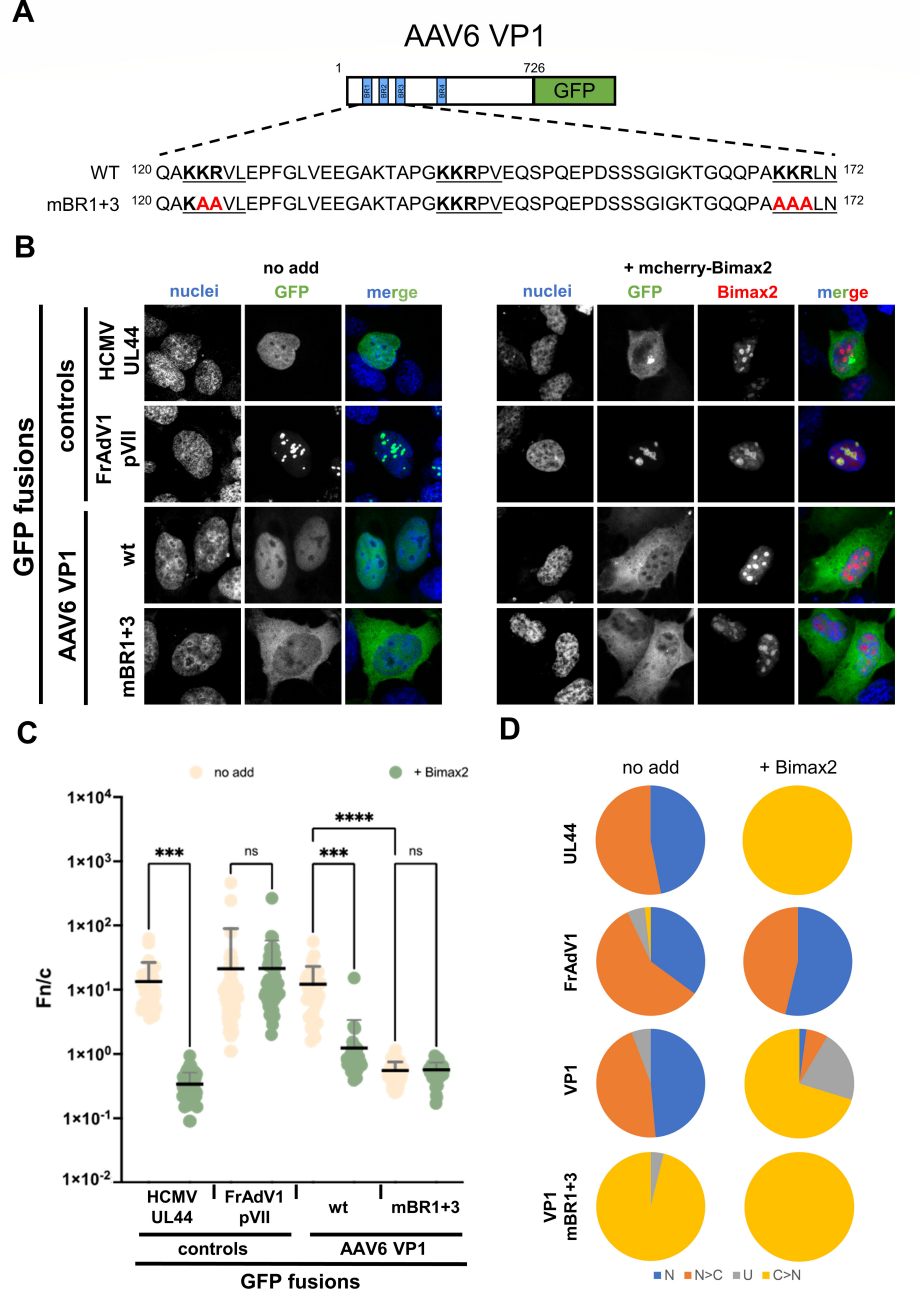
AAV6 VP1 nuclear localization is mediated by the IMPα/IMPβ heterodimer. (**A**) HEK 293A cells were seeded on glass coverslips and transfected to express the indicated GFP fusion proteins in the presence or absence of mcherry-Bimax2. Sequences include mutated residues in red. (**B**) Twenty-four hours later, cells were treated with DRAQ5 to stain cell nuclei, fixed, and mounted on microscope slides to allow quantitative CLSM analysis. Representative images of the indicated GFP fusion proteins expressed in the absence (left panels, no add) or presence (right panels, +mcherry-Bimax2) of mcherry-Bimax2. Images of the indicated channels are shown, along with a merged image. (**C**) Micrographs such as those shown in panel** B **were quantitatively analyzed to calculate Fn/c relative to each GFP fusion protein at the single-cell level. Individual measurements are shown, along with the mean (black horizontal bars) and standard deviation of the mean (gray vertical bars) from three independent experiments. Results from the Welch and Brown-Forsythe ANOVA test of significance are shown. ***P < 0.0005, ****P < 0.0001. (**D**) The percentage of cells relative to each indicated GFP fusion protein, displaying the indicated subcellular localization, is shown. N, nuclear, Fn/c  ≥ 10; N  >  C, nuclear more than cytosolic, 2  ≤  Fn/c  <  10; U, ubiquitous, 1  ≤  Fn/c  <  2; C  >  N, more cytosolic than nuclear, Fn/c  <  1. ns, non-significant.

Since the AAV6 VP1-BR (residues 115–174) directly binds IMPα isoforms but not IMPβ1, we hypothesized that AAV6 VP1 nuclear import is dependent on the classical IMPα/IMPβ import pathway. We set out to address this by measuring the effect of Bimax2, a well-characterized inhibitor of IMPα (74, 93, 95), on nuclear accumulation of full-length AAV6 VP1. To this end, HEK 293A cells were transfected to transiently express full-length AAV6 VP1 fused to GFP (Fig. 3A), either alone or in the presence of mcherry-Bimax2. Subsequently, its levels of nuclear accumulation were quantitatively analyzed by CLSM (Fig. 3B and C). HCMV UL44, which is transported into the nucleus by the IMPα/IMPβ heterodimer (71), and frog adenovirus (FrAdV1) pVII, which is transported into the nucleus via multiple pathways (72), were used as positive and negative controls for Bimax2 response, respectively.

As expected, in the absence of mcherry-Bimax2, HCMV UL44 localized in the nucleoplasm but was excluded from the nucleoli, whereas FrAdV1 pVII mainly localized at the nucleolar level (Fig. 3B). Both proteins strongly localized in the cell nucleus in the majority of transfected cells (mean Fn/c 13.4 and 21.2, respectively; see Fig. 3C). Importantly, in the presence of mcherry-Bimax2, UL44 nuclear localization was significantly impaired (mean Fn/c 0.3), and the protein localized more in the cytosol than in the nucleus in 100% of cells co-expressing Bimax2 (Fig. 3D). On the other hand, FrAdV1 pVII localization was not affected by Bimax2, and the protein accumulated in the nucleus in 100% of cells co-expressing Bimax2 (Fig. 3D), with a mean Fn/c of 21.4 (Fig. 3C).

As expected, in the absence of mcherry-Bimax2, AAV6 VP1 accumulated in the cell nucleus (Fig. 3B) in >90% of transfected cells (Fig. 3D), with a mean Fn/c of 12.2 (Fig. 3C). Importantly, co-expression with mcherry-Bimax2 strongly impaired its nuclear localization (Fig. 3B), resulting in AAV6 VP1 accumulating in the nucleus in only ~5% of cells (Fig. 3D), with a mean Fn/c of 1.24. We then set out to determine if the identified bipartite NLS observed in our structural data and biochemical assays was driving nuclear import of full-length AAV6 VP1. The key binding determinants resolved in our structure were mutated to Ala residues, including minor binding site residues Lys^123^ and Arg^124^ and major binding site residues Lys^168^, Lys^169^, and Arg^170^ (Fig. 3A). This construct is referred to herein as AAV6 VP1 mBR1+3. Subsequently, we quantified the levels of nuclear accumulation of AAV6 VP1 mBR1+3 fused to GFP by CLSM, in the presence and absence of mcherry-Bimax2. Inactivation of the AAV6 VP1 bipartite NLS completely abolished efficient nuclear accumulation, regardless of the presence of Bimax2 (Fig. 3B). AAV6 VP1 mBR1+3 did not accumulate in the nucleus of any cell (Fig. 3D), with a mean Fn/c of approximately 0.6 in the presence and absence of Bimax2 (Fig. 3C). Therefore, AAV6 VP1 is translocated into the nucleus by the IMPα/IMPβ heterodimer due to recognition of a bipartite NLS located in its N-terminus, thus following the classical nuclear import pathway.

## DISCUSSION

In this study, we explored the molecular interactions between the N-terminal AAV6 VP1-BR and nuclear import receptor proteins, with the aim to discern the specific interactions guiding the nuclear entry process. Utilizing both EMSA and FP assays, we first compared binding of AAV6 VP1-BR with IMPα isoforms and IMPβ1, revealing affinity for all tested IMPα isoforms, across subfamilies SF1, SF2, and SF3, but excluding IMPβ1. This marked preference for IMPα over IMPβ1 is typical of proteins transported into the nucleus via the classical nuclear import pathway, consistent with findings from prior studies on different AAV serotypes (30–32). Such hypothesis was confirmed by quantitative CLSM analysis of the subcellular localization of AAV6 VP1 in mammalian cells, with the IMPα/IMPβ inhibitor Bimax2 strongly inhibiting nuclear accumulation. These data emphasize the potential universality of the IMPα pathway for AAV viral nuclear import, a crucial aspect for gene therapy applications (30–32). This pattern suggests a strategic adaptation of AAVs to exploit the IMPα pathway for efficient nuclear entry, meriting further experimental validation to fully understand its implications for gene therapy.

Our quantitative investigation revealed a distinct binding hierarchy of AAV6 VP1-BR with IMPα isoforms, showing strongest affinity for IMPα5 and IMPα7 from the SF3 subfamily. In comparison, SF1 members (IMPα1/IMPα2) exhibited lower affinity, whereas IMPα3 from SF2 displayed intermediate affinity. This specificity of AAV6 VP1-BR for a particular IMPα subfamily has not been described previously. Since the efficacy of cargo nuclear import correlates with its affinity for transport receptors (98), AAV6 may exhibit optimized nuclear import in tissues with a high presence of these specific import receptors, opening new avenues for targeted gene therapy applications.

AAV6 displays tissue tropism for skeletal muscle (99–101), airway epithelial cells (102, 103), liver cells (101, 104), and cardiomyocytes (101, 105–107), and is actively studied in numerous pre-clinical and clinical trials (103, 108–111). Particular IMPα isoforms exhibit higher expression levels in specific tissues (112–116), which could be considered in the design of chimerically engineered AAV capsids to improve nuclear localization efficiency of AAV therapy vectors. For example, the N-terminal BR region of an AAV serotype whose NLS has a low affinity for IMPα could be replaced with an NLS with a high affinity for IMPα. In the case of AAV6, which has a moderate affinity for IMPα, replacing its NLS with that from an AAV serotype with a much higher affinity for IMPα may result in increased transduction efficiency. VP1 from a porcine serotype of AAV was previously found to be a very strong binder of IMPα isoforms, with the weakest affinity calculated at a *K*_D_ of 1.0 nM (31), a ~24-fold increase in binding strength compared to that of the strongest AAV6 VP1-BR binder with a *K*_D_ of 24 nM. Theoretically, replacing the NLS region of AAV6 VP1 with that of the porcine AAV VP1 could increase uptake of the AAV vector into the nucleus and subsequently increase transgene expression and therapeutic effect.

In a 2021 study (117), chimeric AAV vector capsids were formed, utilizing different regions of VP1/VP2/VP3 from various AAV serotypes. It was found that AAV6 capsids could be formed using the N-terminal regions of VP1 and VP2 of AAV4, AAV2, and AAV12. These chimeras increased the efficiency of transduction, while variants utilizing only the VP1 unique (region not held by VP2 or VP3, includes BR1) region of alternative AAVs with the VP2 N-terminus of AAV6 did not. This showed that the N-terminal domain of AAV VP1/VP2 can have a huge impact on the way AAV capsids can transduce cells and enter the nucleus for genomic integration. It also supports evidence from this study that BR3 (found in both VP1 and VP2) is a likely contributor to nuclear localization and possibly overall transduction of AAV6. Considering this, it is possible that merely changing the ~60 residues that form the NLSs of AAVs could improve nuclear transduction and subsequent transgene expression without losing the tissue specificity needed for therapeutic targeting. Further, engineering these sections of vectors alone will not affect cell receptor binding or the PLA2 domains that play a critical role in endosomal release of the vector prior to nuclear import. Strength of NLS binding to IMPα does have the potential to prevent release of cargo if it can outcompete the autoinhibitory function of the IMPα IBB domain. The IMPα/IMPβ complex is dissociated when RanGTP binds IMPβ1, releasing the IBB domain of IMPα, which then binds back onto the IMPα binding groove and releasing the cargo NLS and subsequently the cargo itself. A balance between NLS binding strength and adequate release from IMPα must be determined, as theoretically, with high enough affinity the IBB domain can be outcompeted. However, if a balance is met, this has the potential to open up a whole new way of improving recombinant AAV efficiency that may allow for an adjustment to dosage size or even the use of AAV strains found in other animals that humans do not have pre-existing antibodies for.

Through structural analyses, we identified specific regions and residues within AAV6 VP1 that serve as a bipartite NLS, facilitating interaction with host IMPα for nuclear translocation via the classical nuclear import pathway. In this pathway, IMPα functions as an adaptor, binding IMPβ1 for nuclear entry through the nuclear pore complex. Our findings add to a body of evidence (30–32) showing AAV VP1 NLSs can interact with IMPα as either classical monopartite or bipartite NLSs. This diversity, as well as its impact on nuclear import efficiency, warrants further exploration.

The binding mode of AAV6 VP1-BR with host IMPα is consistent with that observed for other bipartite NLS cargoes, engaging both the minor (ARMs 6–8, P1′–P3′) and major (ARMs 2–4, P1–P5 sites) binding sites of IMPα (92), with AAV6 VP1 BR1 and BR3 bound in the minor and major sites, respectively. AAV6 VP1 demonstrated similar NLS binding characteristics to those seen in other AAV studies (30–32). Its ability to bind IMPα, inability to bind IMPβ1, and binding preferences for different IMPα isoforms suggest that BR1 and BR3 drive the interactions between AAV6 VP1 and host import proteins. Nuclear localization of the capsid is critical for AAV function as a gene therapy vector, with the ability to traverse the nuclear membrane and release a transgene for subsequent translation of therapeutic proteins being an essential phase. Until recently, it had been merely assumed that AAVs must contain an NLS in these BRs and bind host import proteins to translocate through the nuclear pore complex. Our cellular data have confirmed that AAV6 VP1 uses the IMPα/IMPβ classical pathway for nuclear import, as well as validated the exact binding mechanisms proposed in our structural and biochemical data. It clearly shows that full-length AAV6 VP1 uses BR1 in conjunction with BR3 as a bipartite NLS to facilitate trafficking into the cell nucleus and that without these clusters, nuclear localization is prevented. This is the first study to determine the precise mechanisms utilized by full-length AAV6 VP1 to translocate to the cell nucleus.

Lastly, it should be noted that despite the evidence put forth by this study, there may be differences in VP1:importin interactions occurring at the cellular level in the context of infectious AAV capsid complexes. However, the fact that the AAV VP1 N-terminal domain is a largely disordered flexible region that becomes exposed prior to nuclear localization (49–51, 55) strongly suggests that BR1 and BR3 act as the NLS for this important stage of the AAV transduction process.

## Data Availability

Maps and model for AAV6 VP1-BR in complex with IMPα have been deposited to the Protein Data Bank, under accession code 9CFT.
